# A Bayesian genome screening of maximum number of drinks as an alcoholism phenotype with the new Haseman-Elston method

**DOI:** 10.1186/1471-2156-6-S1-S116

**Published:** 2005-12-30

**Authors:** Cheongeun Oh, Shuang Wang, Nianjun Liu, Liang Chen, Hongyu Zhao

**Affiliations:** 1 Department of Epidemiology and Public Health, Yale University School of Medicine, New Haven, CT 06520 USA; 2 Department of Biostatistics, Department of Preventive Medicine, University of Medicine and Dentisry of New Jersey, Newark, NJ 07101, USA; 3 Department of Biostatistics, Mailman School of Public Health, Columbia University, New York, NY 10032 USA; 4 Department of Biostatistics, University of Alabama at Birmingham, Birmingham, AL 35294, USA; 5 Department of Molecular, Cellular and Developmental Biology, Yale University, New Haven, CT 06520 USA; 6 Department of Genetics, Yale University, New Haven, CT 06520 USA

## Abstract

Common human disorders, such as alcoholism, may be the result of interactions of many genes as well as environmental risk factors. Therefore, it is important to incorporate gene × gene and gene × environment interactions in complex disease gene mapping. In this study, we applied a robust Bayesian genome screening method that can incorporate interaction effects to map genes underlying alcoholism through its application to the data of the Collaborative Studies on Genetics of Alcoholism provided by Genetic Analysis Workshop 14. Our Bayesian genome screening method uses the regression-based stochastic variable selection, coupled with the new Haseman-Elston method to identify markers linked to phenotypes of interest. Compared to traditional linkage methods based on single-gene disease models, our method allows for multilocus disease models for simultaneous screening including both main and interaction (epistatic) effects. It is conceptually simple and computationally efficient through the use of Gibbs sampler. We conducted genome-wide analysis and comparison between scans based on microsatellites and single-nucleotide polymorphisms. A total of 328 microsatellites and 11,560 single-nucleotide polymorphisms (by Affymetrix) on 22 autosomal chromosomes and sex chromosome were used.

## Background

Alcohol dependence is a complex disorder that is influenced by many genetic and environmental factors. Identifying genes associated with alcohol dependence is critical to understand its etiology and to develop efficient methods for prevention and treatment. However, this effort has been hampered by the complexity underlying alcohol dependence: rather than there being one or a few major genes affecting alcohol dependence, it is likely that multiple genes interact with each other, together with environmental factors, to affect susceptibility to alcohol dependence. In this paper, we describe analyses of the Collaborative Study on the Genetics of Alcoholism (COGA) data (Problem 1), using self-reported "maximum number of drinks consumed in a 24-hour period" (denoted by M) as a quantitative trait, to map genes underlying alcohol dependence. The measure M is closely related to alcoholism diagnosis and provides a quantitative measure for alcohol dependence. For genome screens for this trait, we use the modified Haseman-Elston regression method [1] along with the Bayesian variable selection methods developed by Oh [2] to locate susceptibility genes for alcohol dependence and assess their epistatic effects.

The Haseman-Elston method and its derivatives allow one to apply linear regression methods in linkage analysis. For each sibling pair, these methods use the number of alleles identical by descent (IBD) at each marker as the explanatory variable and a statistic measuring similarity of the quantitative traits in the sibling pair, squared difference, or cross-product, as the response variable.

In practice, the number of markers and their possible epistatic effects are often larger than the number of observations (patients or sib-pairs), where the design model is referred as being "supersaturated". As we often have hundreds of markers to consider, we must deal with the problem of multiple testing in this context. Besides, if one would like to take epistasis into account, the number of tests can easily exceed tens of thousands. Performing hypothesis tests for linkage for all of these possibilities without appropriate adjustment of multiple comparisons can lead to the identification of spurious genetic effects or the masking of real effects. The supersaturated nature of the design model also makes the conventional best subset model selection methods [3] practically infeasible. To overcome this infeasibility, efficient and robust Bayesian variable selection methods for multiple regressions were proposed by Oh [2] utilize the stochastic search variable selection (SSVS) methodology developed by George and McCulloch [4] in gene mapping. SSVS uses Gibbs sampling to sample from the posterior distribution of the model space and obtain the "best" subsets from the sampled posteriors. Therefore, it is capable of considering a large number of candidate variables without evaluating all possible models. Oh et al. [5] applied this approach to genetic linkage studies and successfully estimated main and epistatic effects. George and McCulloch's method [4], yet, is known to be sensitive to the choice of the priors. Oh [2] modified and extended SSVS, focusing only on statistics robust to the choice of the prior. These methods were shown to be computationally more efficient than the original SSVS [4] in handling many hundreds of thousands epistatic effects and gene × gender interactions through the simulation study by Oh [2].

In this study, we apply the method developed by Oh [2] to the COGA data of Genetic Analysis Workshop 14 (GAW14) to screen disease susceptibility genes related to the quantitative phenotype, "maximum number of drinks consumed in a 24-hour period", and compare the results from single-nucleotide polymorphisms (SNPs) with those from microsatellites. Because SNPs offer denser and more automated genotyping than microsatellites, we expect that SNP-based linkage analysis may have better performance in the mapping of disease loci.

## Methods

### Haseman-Elston method

The original Haseman-Elston method [6] is a general model-free method for testing linkage between candidate markers and quantitative trait loci on a sample of sib-pairs. This method involves the regression of the squared trait difference *D*^2 ^= (*X*_1 _- *X*_2_)^2^) in pairs of siblings on the number of alleles shared IBD between each sib pair at a given marker. Although the original Haseman-Elston method is simple, robust, and computational inexpensive, it may ignore information contained in the observed bivariate data. In fact, the squared mean corrected trait sum of sib pairs (*S*^2 ^= (*X*_1 _+ *X*_2 _- 2*μ*)^2^) may provide additional information on the genetic effect [7]. This observation has led to a number of methods to modify the original Haseman-Elston method by combining both *D*^2 ^and *S*^2 ^in linkage analysis to improve statistical power. One of the simplest methods was proposed by Elston et al. [1], which uses *CP *= (*S*^2 ^- *D*^2^)/4 = (*X*_1 _- *μ*)(*X*_2 _- *μ*) as the response variable in the regression. It essentially averages both regression coefficient estimates (of *D*^2 ^and *S*^2^) with equal weights. An additional advantage of using CP as the response variable is that it may be more normally distributed than *D*^2 ^and *S*^2^.

### Bayesian genome screening

Assume that we observe *m *markers along the genome. Among these *m *markers, some may display some evidence of tight linkage to genes with large effects and others only with weak effects. They may exhibit main effects and/or epistatic effects. In our regression setup, the *CP*s are treated as the responses and the IBD status at each marker *X*_*j *_is the candidate explanatory variable. Then the observed *CP *value of sib-pair *i *can be described by the following linear model



where *n *is the number of sib pairs and the errors *ε*~N(0, σ^2^) are assumed to be independent. A subset model is represented by a binary vector *γ* = (*γ*_1_, ..., *γ*_*m*_), where *γ*_*j *_= 1 or *γ*_*j *_= 0 represents the presence or absence of variable *j *in the model. A large effect of *α*_*j *_indicates that marker *j *has the evidence for linkage and  indicates the epistatic effect between markers *j*_1 _and *j*_2_. The marker effects *α*_*j *_(*j *= *1*,..., *m*) are given the prior of the mixture of normal and point-mass distributions conditional on the indicators *γ*_*j*_, *α*_*j*_|*γ*_*j*_~*γ*_*j*_*N*(0,*c*^2^) + (1 - *γ*_*j*_)*δ*_*o*_. Similarly, the epistatic effects (*j*_1_, *j*_2 _= *l*,..., *m*) with *j*_1 _<*j*_2 _are given . Hence, if the effect is absent in the model choice (*γ*_*j *_= 0), we can simply omit that factor when building the model. If *γ*_*j *_= 1, the magnitude of the effect is large and then a nonzero estimate should be included in the model and its posterior distribution is largely determined by the data. The prior *γ *should represent beliefs about the relationship between factors. If only main effects but no epistatic effects are considered, the prior of *γ *can be simply set as prob(*γ*) = *p*^*k*^, where *k *is the number of ones in *γ*. However, when epistatic effects are considered in model selection, the model space becomes enormous and the independent relationship may not be the proper assumption anymore. In this case, we incorporate Chipman's [8] hierarchical prior structure into our model space. Then the probability that the term  is present  may take on four different values, depending on the values of the pair ,



The choice of the values, (*p*_00_,*p*_01_,*p*_10_,*p*_11_) represents the belief of the relationship between factors  (main effects), and  (epistatic effects). If we believe that an epistatic effect without main effects is quite unlikely, we can simply set the prior as (*p*_00_,*p*_01_,*p*_10_,*p*_11_) = (0,0,0,*p*). Alternatively, we can use (*p*_00_,*p*_01_,*p*_10_,*p*_11_) = (0,*p*_1_,*p*_2_,*p*_3_) to relax these conditions.

With an appropriate prior distribution on σ^2^, one can obtain the posterior distribution of *γ *using Gibbs sampling. Then, by examining the posterior distribution of *γ*, one can identify the optimal model with the highest posterior. However, since our interest is to find the markers having evidence for linkage, we proceeded to obtain the marginal posterior probabilities of different factors and rank their relative frequencies. That is, the marginal posteriors of all main effects and epistatic effects are obtained and used to rank these effects. We view the ranking as a measure of relative importance of all the factors, i.e., the degree of evidence for these factors linked to the disease genes. Our previous studies showed that the rankings of these factors are very robust [2], especially for those with the highest marginal probabilities, which are the focus of our study. Probabilities much less than 0.5 for both main and epistatic effects represent the belief that relatively few terms are active. This assumption is quite reasonable for our applications of gene screening, because there are only a few markers linked to the disease genes out of hundreds or thousands of markers. Even though the choice of *p *may be arbitrary, the results are robust to its setting. We should note that the priors for *p *are chosen partly to facilitate the computing.

In microsatellites and SNPs, there are 328 and 7,826 markers considered, respectively. Therefore, to consider epistatic effects, we needed to include 53,957 factors for microsatellites and about 30 millions factors for SNPs in the models. We set the prior as *p *= 0.05 for microsatellies and *p *= 0.005 for SNPs. There are about 1,499 sib pairs in the overall sample. We use 1,433 sib pairs for microsatellites analysis and 1,322 sib pairs for SNPs analysis in our study. The number of alleles shared IBD is obtained using GENEHUNTER (v.2.1) for microsatellites and MERLIN for SNPs for each sib pair at each marker. Because it is impractical to track the complete posterior of *γ*, only the marginal posterior of each marker is obtained. In our analysis, we use (*p*_00_,*p*_01_,*p*_10_,*p*_11_) = (0,*p*,*p*,*p*) to give all the factors an equal opportunity to be included in the final model as long as one of main effects is in the model.

## Results

In the full sample of cases, the quantitative trait M ranges from 0 to 160. Five individuals are in the highest threshold class (M > 128 drinks). The highest reported M is 160. The use of the log transformation minimizes their impact on the regression analysis, which can be inflated by self-report [9]. As reported in Saccone [9], there is a close relationship between diagnosis and M.

**Figure 1:**
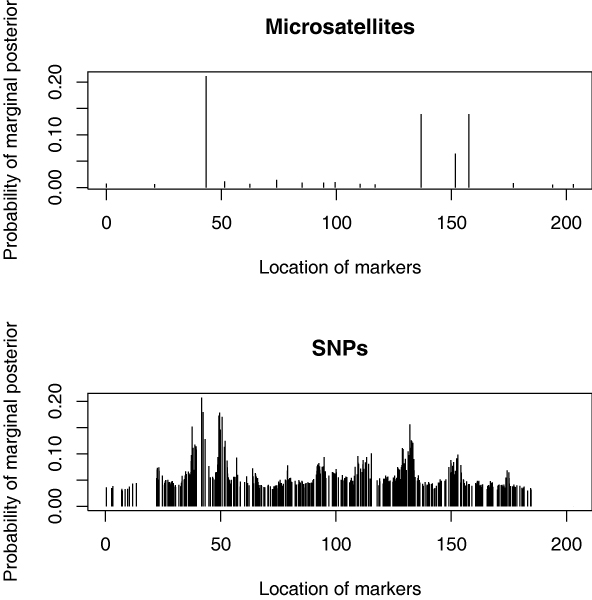
**Comparisons between microsatellites and SNPs on chromosome 4**. Both results from microsatellites and SNPs show similar patterns for markers having the evidence being linked to the disease genes.

In each analysis, the Markov chain Monte Carlo (MCMC) sampler was run for 100,000 cycles after discarding the first 2,000 cycles for the burn-in period. Because MCMC samplers arise from recursive draws, they produce correlated samplers from the posteriors. Therefore, the chains are thinned (one iteration in every 10 cycles is saved) to reduce serial correlation in the stored samples. The total number of samples kept in the post-Bayesian analysis is 10,000. It takes ~2 hours for microsatellites and ~6 hours for SNPs to generate each sample with JAVA programs on a Linux cluster using 2.4-GHz Intel processors. Table 1 displays the rankings of the marginal posteriors for the main effect and epistatic effect screening obtained from microsatellites and SNPs. Table 1 clearly shows that the high posteriors comparatively concentrate on chromosome 4 both for microsatellites and SNPs, which agrees with Saccone [9]. To localize the specific regions of the strong evidence for linkage on chromosome 4, we extract the relative frequencies of the marginal posteriors. Figure 1 shows the comparisons of SNPs vs. microsatellites in the regions having evidence of linkage, and they have similar patterns. For gene × gene interaction, microsatellites are able to locate epistatic effects between chromosomes 8 and 15, and between chromosomes 10 and 17, whereas SNPs locate epistatic effects only between sex and markers on chromosome 23 (sex chromosome). However, the marginal posterior probabilities are not strong enough to support the evidence of epistatic effects.

## Discussion

In this study, we have compared the genome-wide linkage analyses based on microsatellites and SNPs. Our methods located the main effects of markers both from microsatellites and SNPs and produced similar patterns between them. However, the results for epistatic effect screening are less consistent and revealing. This might be purely because these epistatic effects are weak in nature and further research in this area is warranted.

## Conclusion

Bayesian genome screening methods provide a powerful and efficient tool in identifying potential markers and their epistatic effects. They are very effective because they are able to conduct searches over the entire model space; while the frequentist's best subset model selection procedure is constrained by computing power required to examine all candidate models. In addition, Bayesian genome screening methods can work on problems with many more candidate variables, which is essential to consider when epistatic effects are studied. When one tries to locate the epistatic effects, the number of covariates (factors) easily far outnumbers the sample size. Most traditional linkage methods do not work under this condition because they often assume a single-gene model and test effects one at a time. By using the prior structures that reflect the relationship among the candidate variables, our general approach can accommodate a large number of candidate markers as well as their epistatic effects by evaluating all factors simultaneously. We were able to locate markers on chromosome 4 that show the strong evidence of linkage with alcoholism related to quantitative phenotype, "maximum number of drinks consumed in a 24-hour period", both from microsatellite and SNP scans and weak evidence for epistatic effects.

## Abbreviations

COGA: Collaborative Study on the Genetics of Alcoholism

GAW14: Genetic Analysis Workshop 14

IBD: Identical-by-descent

MCMC: Markov chain Monte Carlo

SNP: Single-nucleotide polymorphism

SSVS: Stochastic search variable selection

## Authors' contributions

CO participated in the design of the study, performed the analysis, and drafted the manuscript. SW helped to obtain IBD values for linkage analysis. SW, NL, LC, and HZ participated in the design and the discussion of the study, and the preparation of the manuscript. All authors read and approved the final manuscript.

## Figures and Tables

**Table 1:** Comparisons of SNPs and microsatellites for main effect and two-way interaction effect screening. Both microsatellite and SNP analyses show a strong and frequent main effect in chromosome 4, whereas epistatic effects are located differently.

Ranking	Chromosome	Marginal posterior probabilities
Microsatellites		
1	Chr 4	0.21133
2	Chr 6, Chr 13, Chr 16	0.15433
3	Chr 4 (2 markers^a^), Chr 10	0.13922
4	Chr 23, Chr 17, Chr 7	0.09066
5	Chr 23, Chr 2	0.08533
6	Chr 1	0.08466
7	Chr 13	0.07577
8	Chr 16	0.07422
9	Chr 14 (2 markers)	0.07266
10	Chr 7	0.06944
11	Chr 17	0.06922
12	Chr 3	0.06622
13	Chr 20	0.05766
14	Chr 8 × Chr 15^b^	0.05644
15	Chr 10 × Chr 17^b^	0.03244
SNPs		
1	Chr 4	0.2068
2	Chr 4 (2 markers^a^)	0.1793
3	Chr 23	0.1786
4	Chr 4	0.1725
5	Gender	0.1703
6	Chr 3, Chr 13	0.1563
7	Chr 23	0.1516
8	Chr 23 × sex^b^	0.1461
9	Chr 23	0.1295
10	Chr 4, Chr 6	0.128
11	Chr 6, Chr 23	0.1256
12	Chr 3	0.1247
13	Chr 16	0.1237
14	Chr 7, Chr 4	0.1208
15	Chr 14	0.0209

^a^Two markers are ranked.

^b ^Epistatic effect between the two chromosomes
